# 기혼 폐경 여성의 주관적 건강상태 및 부부동반활동과 우울의 관련성: 여성가족패널조사 9차년도 자료의 상관성 연구

**DOI:** 10.4069/whn.2026.09.15

**Published:** 2026-09-29

**Authors:** Ee Jee Jung, Yee Jung Jang, Ji Seon Lee

**Affiliations:** Department of Nursing Science, Ewha Womans University, Seoul, Korea; 이화여자대학교 일반대학원 간호과학과

**Keywords:** Depression, Health status, Postmenopause, Spouses, Women

## Abstract

**Purpose:**

Using data from the ninth wave of the Korean Longitudinal Survey of Women and Families (KLoWF), this study aimed to examine the relationships among subjective health status, spousal shared activities, and depression and to identify factors associated with depression among married postmenopausal women.

**Methods:**

A cross-sectional secondary analysis was performed using data from 2,317 married postmenopausal women aged 40–64 years in the ninth wave (2023) of KLoWF. SPSS/WIN 30.0 was used to calculate descriptive statistics and Pearson correlation coefficients and to conduct one-way analysis of variance and hierarchical multiple regression analyses.

**Results:**

Depression was negatively correlated with subjective health status (r=−.35, *p*<.001) and spousal shared activities (r=−.05, *p*=.017). Subjective health status and spousal shared activities were positively correlated (r=.12, *p*<.001). Hierarchical multiple regression identified a significant association between subjective health status and depression (β=−.31, *p*<.001), but no significant association between spousal shared activities and depression (β=.02, *p*=.257). The final model had an adjusted R^2^ of .14. In exploratory analyses, depression was positively associated with watching movies, performances, and sports events with a spouse (β=.08, *p*<.001); none of the other shared activities had a significant association with depression.

**Conclusion:**

Subjective health status had the strongest association with depression among married postmenopausal women. The findings suggest that interventions to promote mental health in this population should take subjective health status into account. Further research is needed to determine how associations with mental health outcomes vary by the specific type of spousal shared activity.

## Introduction

여성의 평균 기대수명이 증가함에 따라 폐경 이후의 삶은 여성 생애주기의 상당 부분을 차지하게 되었다. 한국 여성의 자연 폐경은 일반적으로 50세 전후에 발생하는 것으로 알려져 있으며[1], 평균수명의 증가에 따라 여성은 생애의 상당 기간을 폐경 이후 상태로 보내는 것으로 나타났다[2]. 폐경은 단순한 생리적 변화가 아니라 신체적 기능 저하, 감정 변화, 대인관계의 재조정 등 다양한 변화를 동반하는 복합적 과정이다[3]. 특히 여성이 한 가정의 중심축이 되어 가족 구성원들에게 중요한 역할을 하는 시기에 발생하므로[4], 폐경 여성의 건강 문제는 개인뿐 아니라 배우자 및 가족과의 관계에도 영향을 미칠 수 있다[5]. 따라서 폐경 여성이 건강한 상태를 영위할 수 있도록 돕는 것은 개인과 가족의 삶의 질 유지를 위해 매우 중요하다.

폐경 여성의 우울에 영향을 미치는 주요 개인 내적 요인 중 하나는 주관적 건강상태이다. 주관적 건강상태는 객관적 건강지표로 측정하기 어려운 개인의 신체적·정신적 건강 수준을 포괄적으로 반영하는 지표이며[6,7], 우울과 유의한 관련성이 있는 것으로 보고되었다[8-10]. 특히 주관적 건강상태가 낮을수록 우울 수준이 높은 것으로 나타났다[9,10]. 이는 자신의 건강을 부정적으로 인식하는 것이 정서적 취약성을 증가시키고 정신건강에 부정적인 영향을 미칠 수 있음을 시사한다.

폐경 여성의 건강은 개인의 건강 인식뿐 아니라 주변 관계 및 환경과 같은 관계적 요인의 영향도 받는 것으로 알려져 있다. 기혼 여성의 배우자 관계는 중년기 여성의 심리적 적응 및 우울과 관련된 중요한 관계적 요인으로 보고되고 있으며[11,12], 배우자와 함께 참여하는 부부동반활동에서 여가∙사회∙가족 활동은 폐경 여성의 정서적 안정 및 우울과 관련되는 관계적 요인으로 주목받고 있다[13]. 기존 연구에서 주로 다룬 결혼 만족도나 배우자 지지가 부부관계에 대한 주관적 평가 또는 정서적·도구적 지원을 반영하는 개념이라면, 부부동반활동은 배우자와 함께 여가·사회·가족 활동에 참여하는 구체적인 행동적 상호작용을 반영한다[13,14]. 특히 부부동반활동은 일상생활에서 관찰하고 변화시킬 수 있는 활동 특성이라는 점에서, 폐경 여성의 관계적 건강요인을 사정하고 부부 단위의 간호중재 전략을 개발하는 데 활용 가능한 변수로 고려할 수 있다[13]. 반면 배우자와의 교류가 부족하거나 관계적 상호작용이 제한된 경우 심리적 안녕이 저하될 수 있는 것으로 보고되었다[15].

폐경 여성이 경험하는 다양한 건강문제 중에서도 우울은 정신건강과 삶의 질에 미치는 영향이 크기 때문에 특히 주목해야 할 건강문제이다. 우울은 폐경기 여성의 건강과 관련된 주요 심리적 문제 중 하나이며[16], 선행연구에서는 폐경 이후 여성이 폐경 전보다 우울 증상을 경험할 위험이 더 높은 것으로 보고되었다[17]. 폐경기에 나타나는 호르몬 변화와 이에 따른 다양한 신체적·정서적 증상은 우울을 악화시키는 요인으로 작용할 수 있으며[18], 이는 폐경 여성의 삶의 질 저하와 심리적 고통으로 이어질 수 있다. 또한 우울은 개인의 정서적 안녕을 저해할 뿐만 아니라 건강행위 실천을 감소시켜 실제 건강상태 악화로 이어질 수 있다는 점에서[19], 폐경 여성의 건강증진을 위해 우울과 관련된 요인을 파악하는 것이 중요하다.

지금까지 폐경 여성의 우울과 관련된 선행연구들은 주로 개인 내적 요인인 주관적 건강상태 또는 관계적 요인인 부부관계를 중심으로 이루어져 왔다[10,12]. 그러나 폐경은 신체적·심리적 변화를 동반하는 생애 전환기로, 중년 여성 모두가 동일한 시기에 경험하는 단순한 연령 기반의 변화가 아니며, 폐경 이후 여성은 다양한 신체적·심리적 변화를 경험할 수 있다. 또한 선행연구에서는 동일한 여성가족패널 자료를 활용하여 40–64세 여성의 우울 관련 요인을 분석하였으나[20], 폐경 여부와 결혼 상태를 고려하여 기혼 폐경 여성의 특성에 초점을 둔 분석은 이루어지지 않았다. 특히 부부동반활동은 배우자가 있는 여성의 관계적 특성과 부부 간 상호작용을 반영하는 변수[20]라는 점에서, 기혼 폐경 여성의 우울을 이해하는 데 중요한 관계적 특성으로 고려될 수 있다.

생태체계 이론은 개인의 건강을 개인적 특성뿐만 아니라 가족 및 사회적 관계를 포함한 다양한 환경체계와의 상호작용 속에서 이해하는 관점이다[4]. 국내 기혼 폐경 여성을 대상으로 한 선행연구에서는 이러한 생태체계적 관점을 적용하여 연령, 격렬한 신체활동 및 체질량지수(body mass index, BMI)를 미시체계로, 결혼만족감을 중간체계로, 부부동반 사회봉사활동을 외체계로 분류하고 주관적 건강상태의 변화와 관련 요인을 살펴보았다[4]. 이러한 관점은 기혼 폐경 여성의 건강을 개인적 특성과 관계적 환경을 포함한 다양한 수준에서 이해할 수 있는 이론적 근거를 제공한다.

실제로 개인의 건강 인식을 나타내는 주관적 건강상태는 폐경 여성의 심리적 안녕과 관련된 것으로 보고되었으며[10,21], 부부관계의 맥락을 반영하는 부부동반활동 역시 폐경 여성의 우울과 관련되는 것으로 알려져 있다[13]. 그러나 주관적 건강상태와 부부동반활동을 함께 고려하여 기혼 폐경 여성의 우울과의 관련성을 살펴본 연구는 찾아보기 어렵다. 따라서 기혼 폐경 여성의 우울을 보다 포괄적으로 이해하기 위해서는 개인적 차원의 건강 인식과 관계적 차원의 부부 상호작용을 함께 고려할 필요가 있다. 이에 본 연구는 전국 단위의 대규모 자료인 여성가족패널조사(Korean Longitudinal Survey of Women and Families, KLoWF) 9차년도 자료를 활용하여 기혼 폐경 여성의 주관적 건강상태와 부부동반활동을 단계적으로 고려하여 우울과의 관련성을 위계적으로 분석하고자 한다. 이를 통해 기혼 폐경 여성의 우울과 관련된 개인적·관계적 요인에 대한 이해를 넓히고, 정신건강 증진을 위한 개인 및 부부 단위의 간호중재 전략 개발에 필요한 기초자료를 제공하고자 한다.

본 연구의 목적은 한국여성정책연구원의 여성가족패널조사의 2023년 9차년도 자료를 사용하여 기혼 폐경 여성의 주관적 건강상태, 부부동반활동, 우울의 관계를 확인하고, 우울 수준과 관련된 요인을 파악하는 것이다. 구체적인 연구 목적은 다음과 같다.

(1) 대상자의 일반적 특성과 주관적 건강상태, 부부동반활동, 우울 수준을 파악한다.

(2) 대상자의 일반적 특성에 따른 우울 수준의 차이를 파악한다.

(3) 대상자의 주관적 건강상태, 부부동반활동, 우울 수준 간의 상관관계를 파악한다.

(4) 대상자의 우울 수준과 관련된 요인을 파악한다.

## Methods

**Ethics statement:** This study was approved for exemption from review by the Institutional Review Board of Ewha Womans University (No. ewha-202511-0043-01) because it was a secondary analysis of publicly available, de-identified data from the Korean Longitudinal Survey of Women and Families. The requirement for informed consent was waived because the researchers had no direct contact with participants and no access to personally identifiable information.

### 연구 설계

본 연구는 기혼 폐경 여성의 주관적 건강상태 및 부부동반활동과 우울 간의 관련성을 확인하고, 우울 관련 요인을 파악하기 위해 2023년 여성가족패널조사 9차년도 자료를 이차 분석한 횡단적 상관성 조사연구이다.

### 연구 대상

최종 분석 대상 선정 과정은 Figure 1과 같다. 본 연구는 한국여성정책연구원에서 조사한 여성가족패널조사(Korean Longitudinal Survey of Women and Families)의 9차 조사에 참여한 여성 중 폐경 경험이 있는 기혼여성을 대상으로 하였다. 여성가족패널조사는 여성의 생애주기에 따른 경제활동과 가족생활의 변화를 종단적으로 파악하기 위해 구축된 전국 단위 패널조사이다[22].

본 연구에서는 선행연구[23]를 근거로 폐경 여성의 연령 범위를 만 40–64세로 정의하였다. 이에 2023년 여성가족패널조사 참여자 9,055명 중 연구 대상 연령 기준인 만 40–64세에 해당하지 않는 여성 4,216명을 제외하여, 만 40–64세 여성 4,839명을 선정하였다. 이후 미혼, 이혼, 사별, 별거 및 결혼 상태 무응답자 770명을 제외하여 기혼 여성 4,069명을 선정하였다.

폐경 경험은 여성가족패널조사의 ‘폐경을 경험하셨습니까?’ 문항을 기준으로 확인하였다. 해당 조사에서는 마지막 월경 후 1년이 지난 상태를 폐경으로 정의하였으며, 응답범주는 ‘예’와 ‘아니오’로 구성되어 있다. 본 연구에서는 해당 문항에 ‘예’라고 응답한 경우를 폐경 경험이 있는 것으로 분류하였으며, 원자료에서는 자연폐경, 수술폐경, 약물 또는 질환 관련 폐경을 별도로 구분하지 않았다. 이 중 폐경 경험이 없거나 관련 문항에 무응답 또는 응답을 거부한 1,739명을 제외하여 폐경 경험이 있는 기혼 여성 2,330명을 추출하였다.

연간 가구소득, 본인 교육 수준, 배우자 연령 및 배우자 교육 수준에 결측값이 있는 13명을 제외하여 최종적으로 2,317명을 분석 대상으로 선정하였다(Figure 1).

### 연구 도구

#### 일반적 특성

대상자의 일반적 특성은 연령, 교육 수준, BMI, 주관적 체형 인식, 격렬한 신체활동, 배우자 연령, 배우자 교육 수준, 연간 가구소득 및 결혼만족도를 분석하였다.

연령은 만 나이를 기준으로 측정하였으며, 평균과 표준편차를 산출하고 40–49세, 50–59세, 60–64세로 분류하였다. 교육 수준은 여성가족패널조사에서 초등학교 이하부터 대학원까지의 세부 학제로 조사되었으며, 국내 성인의 우울과 관련한 요인을 분석한 선행연구[24]를 근거로 중학교 졸업 이하, 고등학교 졸업, 대학교 졸업 이상의 3개의 범주로 재분류하였다. 배우자 연령은 만 나이를 기준으로 40-49세, 50-59세, 60-69세, 70세 이상으로 분류하였고, 배우자 교육 수준은 대상자의 교육 수준과 동일한 세부 학제로 조사되었으며, 동일한 기준에 따라 최종 학력을 기준으로 중학교 졸업 이하, 고등학교 졸업, 대학교 졸업 이상으로 재분류하였다[24].

BMI는 체중(kg)을 신장(m)의 제곱으로 나눈 값(kg/m^2^)으로 산출하였다. 대한비만학회의 비만 진료지침에 따라 저체중(<18.5 kg/m^2^), 정상체중(18.5–22.9 kg/m^2^), 과체중(23.0–24.9 kg/m^2^), 비만(≥25.0 kg/m^2^)으로 분류하였다[25]. 주관적 체형 인식은 여성가족패널조사에서 ‘매우 마른 편’부터 ‘매우 살찐 편’까지 5개 범주로 조사되었으며, 집단별 사례 수를 고려하여 마른 편, 보통, 비만인 편의 3개 범주로 재분류하였다[26].

격렬한 신체활동은 여성가족패널조사에서 최근 1주일 동안 평소보다 몸이 매우 힘들거나 숨이 많이 가쁜 신체활동을 수행한 일수를 0일부터 7일까지 조사한 문항으로 측정하였다[22]. 한국의 성인의 신체활동 빈도와 우울과의 관련성을 분석한 선행연구[27]에서 신체활동 일수를 0일, 1–2일, 3–4일, 5–7일의 4개의 범주로 분석하였고, 본 연구에서는 이에 근거하여 0일, 1–2일, 3–4일, 5일 이상의 4개 범주로 재분류하였다[27].

연간 가구소득은 연간 총 가구소득을 기준으로 4분위로 분류하였으며, 1분위는 소득 수준이 가장 낮은 집단, 4분위는 소득 수준이 가장 높은 집단을 의미하며, 한국 성인의 가구소득을 1분위에서 4분위로 구분하여 우울과의 관련성을 분석한 선행연구[26]에 근거하였다. 결혼만족도는 여성가족패널조사에서 ‘남편과의 요즈음 결혼생활에 대한 느낌’을 ‘매우 불행하다’(1점)에서 ‘매우 행복하다’(10점)까지로 평가한 단일 문항이다[22]. 본 연구에서는 원 척도 점수를 기준으로 불만족(1–3점), 보통(4–7점), 만족(8–10점)의 3개 범주로 재분류하였다[28].

#### 주관적 건강상태

주관적 건강상태는 개인이 자신의 전반적 건강 수준을 총괄적으로 스스로 평가하는 개념으로, 질병 유무·증상과 기능상태 등 신체적 정보와 심리·사회적 비교와 기대가 통합되어 형성되는 인지적 자기평가 지표로서[29,30], 본 연구에서는 개인이 지각하는 현재 자신의 전반적 건강 수준에 대한 주관적 평가로 정의하였다[31].

대상자가 스스로 느끼는 건강 정도를 단일 문항으로 측정한 것으로[4], ‘당신의 현재 건강상태는 어떻다고 생각하십니까?’라는 질문에 5점 리커트 척도로 응답하게 하였다. 본 연구에서는 점수를 역환산하여(1점, 매우 나쁘다; 2점, 조금 나쁜 편이다; 3점, 보통이다; 4점, 대체로 좋은 편이다; 5점, 매우 좋다) 점수가 높을수록 주관적 건강상태가 양호함을 나타내도록 하였고, 선행연구에서와 같이 주관적 건강상태 점수를 연속형 변수로 간주하였다[32,33]. 본 연구에서 사용한 주관적 건강상태는 단일 문항으로 측정되므로 신뢰도를 산출하는 데에는 한계가 있었다. 그러나 주관적 건강상태를 측정하는 단일 문항에 대한 신뢰도는 선행연구에서 test–retest reliability를 통해 지지된 바 있으며[34], 이러한 근거를 바탕으로 본 연구에서도 해당 문항을 신뢰성 있는 자기보고 지표로 간주하여 분석에 활용하였다.

#### 부부동반활동

부부동반활동은 배우자와 함께 시간을 보내며 여가나 사회적 경험을 공유하는 관계적 상호작용 과정으로, 단순한 여가활동에 국한되지 않은 다양한 형태의 공동 경험을 의미한다[20]. 본 연구에서 부부동반활동은 배우자와 함께 수행하는 여가, 사회적, 가족적 활동을 의미하며[20], 여성가족패널조사의 부부동반활동 문항을 사용하여 측정하였다. 구체적으로는 ‘영화, 공연, 스포츠 등을 관람하기’, ‘산책, 조깅, 등산, 운동 등을 하기’, ‘사회봉사 및 공동체 참여하기’, ‘시부모님이나 시댁 형제들을 만나기’, ‘친정 부모님이나 친정 형제들을 만나기’로 총 다섯 가지 항목으로 구성되었다. 각 문항은 ‘일주일에 2회 이상’(1점), ‘일주일에 1회 정도’(2점), ‘2주에 1회 정도’(3점), ‘한 달에 1회’(4점), ‘한 달에 1회도 하지 않음’(5점)의 5점 척도로 측정되었다. 본 연구에서는 점수가 높을수록 부부동반활동이 활발함을 의미하도록 각 문항을 역코딩하였다.

여성가족패널조사의 부부동반활동 문항은 활동의 내용은 서로 다르지만 모두 배우자와 함께 수행하는 활동의 빈도를 측정하도록 구성되어 있다. 이에 본 연구에서는 배우자와 함께 수행하는 활동의 전반적인 수준을 반영하는 지표로 활용하기 위하여 동일한 여성가족패널조사의 부부동반활동 문항을 합산하여 지표로 활용한 선행연구[20]를 근거로 5개 문항의 합산 점수를 사용하였다. 다만 각 활동은 여가활동, 신체활동, 사회참여 및 가족교류 등 활동의 특성이 서로 상이하므로 총점은 배우자와 함께 수행하는 활동의 전반적인 수준을 나타내는 지표로 해석하였으며, 이를 보완하기 위하여 활동 유형별 추가 분석을 실시하였다. 총점의 범위는 5–25점이며, 점수가 높을수록 배우자와 함께 수행하는 활동의 전반적인 수준이 높음을 의미한다. 본 연구에서 Cronbach’s α는 .657이었다.

#### 우울

우울은 지속적인 슬픔, 흥미, 상실 등 정서적, 인지적 증상을 특징으로 하는 기분장애로, 생물학적 요인 및 심리사회적 요인이 복합적으로 작용하여 발생한다[35]. 본 연구에서는 Radloff [36]가 개발한 Center for Epidemiologic Studies Depression Scale (CES-D)을 Andresen 등[37]이 10문항으로 축약한 CES-D-10을 여성가족패널조사에서 수정·적용한 문항을 이용하였다. CES-D-10 도구는 지난 1주일간 개인이 느낀 감정이나 태도에 관하여 ‘전혀 없었다’ 0점에서 ‘매일 있었다’의 3점까지로 평가하는 리커트 4점 척도 문항이며, 긍정 문항인 5번과 8번을 역코딩한다[37]. 여성가족패널조사에서는 해당 도구를 ‘대부분 그랬다(일주일 동안 5일 이상)’ 1점에서 ‘극히 드물다(일주일 동안 1일 이하)’ 4점까지로 측정하였다. 따라서 원 척도의 0–3점 채점 체계와 일치시키기 위하여[36,37] 해당 문항의 1점을 0점으로, 4점을 3점으로 조정하며, 긍정 문항인 5번과 8번 문항을 제외한 모든 문항을 역코딩하여 점수를 산출하였다. 본 척도는 0점에서 30점의 범위를 가지며, 점수가 높을수록 대상자의 우울감이 높다는 것을 의미한다. 본 연구에서 Cronbach’s α=.890이었다.

### 자료 수집

본 연구에 사용된 자료는 여성가족패널조사 홈페이지를 통해 자료 사용 승인을 받은 후 CSV 파일 형태로 제공받아 활용하였다.

### 자료 분석 방법

여성가족패널조사의 9차년도 개인 아이디를 기준으로 개인 및 가구 데이터를 병합한 후 IBM SPSS for Windows (ver. 30.0; IBM Corp., Armonk, NY, USA)를 이용하여 분석하였다. 대상자의 일반적 특성, 주관적 건강상태, 부부동반활동 및 우울은 빈도와 백분율, 평균과 표준편차를 이용하여 분석하였으며, 연간 가구소득은 왜도 절대값 2, 첨도 절대값 7을 벗어난 우측 편포를 보였다. 이에 평균이 분포의 중심을 적절하게 대표하기 어려워 중앙값(median)과 사분위범위(interquartile range, IQR)로 제시하였다. 일반적 특성에 따른 우울의 차이는 일원분산분석(one-way analysis of variance, ANOVA)으로 분석하였으며, Levene 검정에서 등분산 가정이 충족되지 않은 경우 Welch ANOVA를 적용하였다. 사후검정은 집단 간 차이가 통계적으로 유의한 변수에 한하여 실시하였으며, 해당 변수는 모두 등분산 가정을 충족하지 않아 Games-Howell post hoc test를 적용하였다. 변수 간 관계는 피어슨 상관계수로 확인하였고, 우울 수준과 관련된 요인은 위계적 다중회귀분석(hierarchical multiple regression analysis)를 이용하여 분석하였다.

위계적 회귀분석은 선행연구와 이론적 근거를 바탕으로 연령, 교육 수준, 연간 가구소득, BMI 및 결혼만족도를 통제변수로 설정하여 실시하였다[12,24,38-40]. 변수의 투입 순서는 폐경 여성의 건강이 개인 내적 수준과 관계적 수준의 요인에 의해 영향을 받는다는 생태체계 이론적 관점[4]에 근거하였다. 1단계에는 통제변수, 2단계에는 주관적 건강상태, 3단계에는 부부동반활동을 순차적으로 투입하였으며, 범주형 변수는 기준집단(reference group)을 설정하여 더미변수화하였다. 회귀모형의 적합성은 공차한계(tolerance), 분산팽창지수(variance inflation factor, VIF) 및 Durbin-Watson 통계량을 이용하여 확인하였다.

주분석 결과의 강건성을 확인하기 위해 추가적인 민감도 분석을 실시하였다. 주분석의 통제변수는 이변량 분석 결과에 따라 선정한 것이 아니므로, 이변량 분석에서 우울과 유의한 차이를 보인 격렬한 신체활동은 주분석의 통제변수에는 포함하지 않았다. 다만, 격렬한 신체활동이 부부동반활동의 신체활동 관련 항목과 밀접하게 관련되어 잠재적 혼란변수로 작용할 가능성을 고려하여, 기존 주분석 모형에 격렬한 신체활동을 추가로 통제하여 주요 결과의 변화를 확인하였다. 또한 결혼만족도는 선행연구[12]에 근거하여 주분석의 통제변수로 포함하였으나, 부부동반활동과 개념적으로 관련될 가능성이 있어[13,14] 결혼만족도의 통제가 부부동반활동과 우울 간의 관련성을 과도하게 조정할 가능성을 고려하였다. 이에 결혼만족도를 제외한 모형을 추가로 분석하여 결혼만족도 통제 여부에 따른 주요 결과의 변화를 확인하였다.

또한 부부동반활동 총점이 우울과 유의한 관련성을 보이지 않아 활동 유형별 독립적인 관련성을 확인하기 위하여 5개 하위 영역을 동시에 투입한 탐색적 회귀분석을 추가로 실시하였다. 모든 통계적 검정의 유의수준은 *p*<.05로 하였다.

본 연구에서는 모집단의 대표 추정치를 산출하는 것이 아니라 분석 대상자 내 변수 간 관련성을 규명하는 것을 목적으로 하였으므로 비가중 분석을 실시하였다. 대규모 국가조사자료를 이용한 2차자료 분석에서는 연구 목적이 모집단 추정보다는 변수 간 관련성을 규명하는 경우 비가중 분석을 사용할 수 있으나, 조사설계가 결과 해석에 미치는 영향을 충분히 고려할 필요가 있다고 제시되고 있다[41]. 복합표본설계를 반영하지 않은 분석은 표준오차 및 통계적 추론에 영향을 미칠 수 있으므로, 본 연구 결과는 모집단에 대한 대표 추정치가 아닌 분석 대상자 내 변수 간 관련성을 중심으로 해석하였다.

## Results

### 대상자의 일반적 특성

평균 연령은 57.12±4.64세였으며, 연령대는 50대가 59.0%로 가장 많았고, 이어 60–64세 35.4%, 40대 5.6% 순으로 나타났다. 교육 수준은 고등학교 졸업이 53.0%로 가장 많았으며, 대학교 졸업 이상 27.6%, 중학교 졸업 이하 19.4%였다. 대상자의 평균 BMI는 23.30±2.70 kg/m^2^였으며, BMI 분포는 정상체중이 47.6%로 가장 많았고, 과체중 29.0%, 비만 21.6%, 저체중 1.7% 순이었다. 주관적 체형 인식은 보통이라고 응답한 대상자가 59.9%로 가장 많았고, 비만으로 인식 31.9%, 마른 편으로 인식 8.2% 순으로 나타났다. 격렬한 신체활동 빈도는 주 0일이 64.6%, 3–4일이 14.2%, 주 5일 이상이 12.7%, 1–2일이 8.5% 순이었다. 배우자의 평균 연령은 60.26±5.61세였으며, 60–69세가 52.7%로 가장 많았고, 50–59세 39.8%, 70세 이상 4.1%, 40–49세 3.3% 순이었다. 배우자 교육 수준은 고등학교 졸업이 45.9%로 가장 많았고, 대학교 졸업 이상 38.5%, 중학교 졸업 이하 15.7% 순이었다. 연간 가구소득의 중앙값은 6,000만 원(IQR, 3,720만 원–8,180만 원)이었으며, 소득 분위는 1분위 24.9%, 2분위 22.8%, 3분위 27.3%, 4분위 25.0%로 비교적 고르게 분포하였다. 결혼만족도는 보통이라고 응답한 대상자가 63.1%로 가장 많았고, 만족 35.0%, 불만족 1.8% 순으로 나타나 전반적으로 결혼 만족 수준은 중간 이상이었다(Table 1).

### 대상자의 주관적 건강상태, 부부동반활동, 우울

기혼 폐경 여성의 주관적 건강상태는 평균 3.38±0.74점(가능 범위 1–5점), 부부동반활동은 6.88±2.50점(가능 범위 5–25점), 우울은 3.51±4.40점(가능 범위 0–30점)이었다(Table 2).

### 대상자의 일반적 특성에 따른 우울

우울은 교육 수준(Welch F=7.69, *p*<.001), BMI (Welch F=4.72, *p*=.003), 격렬한 신체활동(Welch F=3.81, *p*=.010), 연간 가구소득(Welch F=18.41, *p*<.001) 및 결혼만족도(Welch F=44.05, *p*<.001)에 따라 유의한 차이가 있었다. 사후검정 결과, 교육 수준에서 중학교 졸업 이하 집단(4.27±4.80)의 우울이 고등학교 졸업(3.39±4.31) 및 대학교 졸업 이상 집단(3.21±4.22)보다 높았다. BMI에서는 비만군(3.95±4.48)의 우울이 정상체중군(3.18±4.10)보다 높았다. 또한 격렬한 신체활동에서는 활동을 하지 않은 집단(3.65±4.48)의 우울이 주 3–4일 수행한 집단(2.88±3.97)보다 높았다. 연간 소득 수준에서는 1분위 집단(4.54±5.10)의 우울이 2분위(3.55±4.53), 3분위(3.32±4.19) 및 4분위(2.66±3.47) 집단보다 높았고, 2분위와 3분위 집단도 4분위 집단보다 우울이 높았다. 결혼만족도에서는 불만족 집단(8.02±6.39), 보통 집단(3.92±4.69), 만족 집단(2.55±3.37) 순으로 우울이 높았다(Table 3).

### 대상자의 주관적 건강상태, 부부동반활동, 우울 간의 상관관계

주관적 건강상태는 우울과 유의한 음의 상관관계가 있었으며(r=–.35, *p*<.001), 부부동반활동과 우울 간에도 유의한 음의 상관관계가 나타났다(r=–.05, *p*=.017). 주관적 건강상태와 부부동반활동은 유의한 양의 상관관계가 있었다(r=.12, *p*<.001) (Table 4).

### 대상자의 우울 관련 요인

본 연구의 회귀분석 기본 가정을 검토한 결과, 공차한계는 .414–.986으로 모두 0.10 이상이었고, VIF는 1.014–2.415로 모두 10 미만으로 나타나 다중공선성의 문제는 없는 것으로 확인되었다. 또한 Durbin-Watson 통계량은 1.827로 2에 근접하여 잔차의 독립성이 충족된 것으로 나타났다. 따라서 본 연구의 회귀모형은 회귀분석의 가정을 충족하는 것으로 판단하였다.

1단계 모형은 통계적으로 유의하였으며(F=13.40, *p*<.001), 조정된 설명력은 6%였다(adjusted R^2^=.06). 1단계 모형에서 40–49세(β=.05, *p*=.029), 연간 가구소득 2분위(β=–.09, *p*<.001), 3분위(β=–.11, *p*<.001), 4분위(β=–.15, *p*<.001), 저체중(β=.05, *p*=.009), 비만(β=.05, *p*=.015), 결혼 불만족(β=.12, *p*<.001) 및 결혼 만족(β=–.13, *p*<.001)이 우울에 유의한 관련성이 있는 것으로 나타났다. 각 회귀계수는 기준집단(연령 60–64세, 소득 1분위, 정상체중, 결혼만족도 보통) 대비값으로, 연령 60–64세 대비 40–49세와 정상체중 대비 저체중과 비만, 결혼만족도 보통 대비 불만족에서 우울이 높았고, 소득 1분위 대비 2∙3∙4분위와 결혼만족도 보통 대비 만족에서 우울이 낮았다. 한편 본 연구의 통제변수는 이변량 분석 결과가 아니라 선행연구에서 폐경 여성의 우울과 관련이 보고된 변수를 근거로 선정하였다.

주관적 건강상태를 추가로 투입한 2단계 모형도 통계적으로 유의하였으며(F=31.03, *p*<.001), 조정된 설명력은 14%였다(adjusted R^2^=.14). 주관적 건강상태는 우울과 유의한 관련성이 있는 것으로 나타났다(β=–.31, *p*<.001).

부부동반활동을 추가로 투입한 3단계 모형도 통계적으로 유의하였으며(F=28.91, *p*<.001), 최종 모형의 조정된 설명력은 14%였다(adjusted R^2^=.14). 최종 모형에서 주관적 건강상태(β=–.31, *p*<.001)는 우울과 유의하게 관련된 요인으로 나타났으나, 부부동반활동(β=.02, *p*=.257)은 우울에 유의한 관련성이 없었다(Table 5).

주분석 결과의 강건성을 확인하기 위해 추가적인 민감도 분석을 시행하였다. 이변량 분석에서 우울과 유의한 차이를 보였던 격렬한 신체활동을 추가로 통제한 모형에서도 부부동반활동은 우울과 유의한 관련성을 보이지 않았으며(β=.03, *p*=.230), 주분석 결과(β=.02, *p*=.257)와 일관된 결과를 보였다. 격렬한 신체활동의 추가 투입에 따른 설명력의 증가도 통계적으로 유의하지 않았다(ΔR^2^=.003, *p*=.067). 또한 부부동반활동과 개념적으로 관련될 가능성이 있는 결혼만족도의 영향을 확인하기 위해 결혼만족도를 제외하여 분석한 결과에서도 부부동반활동은 우울과 유의한 관련성을 보이지 않았으며(β=.01, *p*=.748), 주분석과 동일한 결론을 보였다.

한편, 부부동반활동 총점은 배우자와 함께 수행하는 활동의 전반적인 수준을 반영하는 지표이므로 각 활동 유형에 따라 우울과의 관련성이 다를 가능성을 고려하여 활동 유형별 추가 분석을 실시하였다. 인구사회학적 통제변수와 주관적 건강상태를 통제한 후, 부부동반활동 총점 대신 5개 하위 영역을 동시에 투입하여 탐색적 회귀분석을 실시하였다. 분석 결과, 영화·공연·스포츠 관람은 우울과 유의한 정적 관련성이 있는 것으로 나타났다(β=.08, *p*<.001). 반면, 산책·조깅·등산·운동(β=–.04, *p*=.054), 사회봉사·공동체 참여(β=.02, *p*=.391), 시댁 가족 만나기(β=.03, *p*=.265), 친정 가족 만나기(β=–.04, *p*=.119)는 우울과 유의한 관련성이 없었다(Supplementary Table 1).

## Discussion

본 연구는 기혼 폐경 여성을 대상으로 주관적 건강상태 및 부부동반활동과 우울 간의 관련성을 확인하고, 이를 바탕으로 폐경 여성의 정신건강 증진을 위한 간호중재 전략 개발에 필요한 기초자료를 제공하고자 수행되었다. 연구 결과, 주관적 건강상태는 기혼 폐경 여성의 우울과 유의한 관련성이 있는 것으로 확인되었으며, 부부동반활동 총점은 최종 회귀모형에서 유의한 관련성을 보이지 않았다. 부부동반활동의 하위 영역별 추가 분석에서는 영화·공연·스포츠 관람만이 우울과 유의한 정적 관련성을 보여, 부부동반활동과 우울의 관계를 이해할 때 활동 빈도뿐 아니라 활동의 유형과 맥락을 함께 고려해야 함을 시사하였다.

본 연구에서 교육 수준, 연간 가구소득, BMI, 격렬한 신체활동, 결혼만족도 등 일부 일반적 특성에 따라 우울 수준에 차이가 나타났다. 이는 낮은 교육 및 소득 수준이 우울과 관련이 있다는 선행연구[10]와 사회경제적 지위가 건강에 영향을 미친다고 보고한 기존 연구[24,40]를 지지하는 결과이다. 또한 결혼만족도에 따른 우울의 차이는 배우자와의 관계의 질이 중년기 여성의 심리적 안정감 및 우울과 밀접하게 관련된다는 선행연구[12,42]와 같은 맥락이다. BMI에 따른 우울의 차이 역시 폐경 후 여성의 우울 관련 요인으로 신체적·건강 관련 특성을 보고한 선행연구[17]와 유사하며, BMI가 개인의 주관적 건강평가와 관련될 수 있음을 보고한 연구[43]와도 연결된다.

구체적으로 본 연구에서는 정상체중군보다 비만군의 우울이 높게 나타났으며, 이는 높은 BMI가 폐경 후 여성의 우울과 정적으로 관련된다는 선행연구[17,39]와 일치한다. 또한 격렬한 신체활동을 하지 않은 집단에서 우울이 높게 나타난 결과는 규칙적인 신체활동이 우울을 완화하는 데 관련된다는 선행연구[44]와도 연결된다. 한편 주관적 건강상태와 부부동반활동 또한 일반적 특성에 따라 유의한 차이를 보여 대상자의 사회인구학적 환경이 개인의 건강 인식 및 부부동반활동 참여와 관련됨을 시사한다. 이러한 일반적 특성은 기혼 폐경 여성의 우울과 관련된 배경 요인으로 고려될 필요가 있으며, 본 연구에서는 이를 통제한 상태에서 주요 변수인 주관적 건강상태와 부부동반활동의 관련성을 확인하였다.

기혼 폐경 여성의 주관적 건강상태, 부부동반활동 및 우울 간의 상관관계를 분석한 결과, 세 변수 간에는 모두 유의한 상관관계가 나타났다. 특히 주관적 건강상태가 높을수록 우울이 낮았으며, 이는 주관적 건강상태와 우울 간의 부적 상관관계를 보고한 선행연구 결과와 일치한다[10]. 또한 위계적 회귀분석에서도 주관적 건강상태는 기혼 폐경 여성의 우울과 유의하게 관련된 주요 요인으로 확인되었으며, 이는 주관적 건강상태가 폐경 여성의 우울을 예측하는 주요 변수라는 선행연구 결과를 지지하였다[17,45]. 폐경 이후 여성은 에스트로겐 감소로 인해 세로토닌과 도파민 등 신경전달물질의 조절에 변화를 경험할 수 있으며, 이는 기분 조절과 밀접하게 관련된다[46]. 또한 폐경기에 경험하는 신체기능 변화, 체중 증가, 수면 변화 및 정서적 변화는 자신의 건강상태에 대한 부정적인 인식으로 이어질 수 있으며, 이러한 인식은 건강행위 실천의 저하와 실제 건강상태의 악화를 초래하여 우울을 심화하는 악순환으로 이어질 가능성이 있다[18]. 이러한 결과는 폐경 여성의 우울을 이해하는 데 있어 객관적인 건강상태뿐 아니라 개인이 인식하는 주관적 건강상태를 함께 고려해야 함을 시사한다. 한편 본 연구에서 우울은 30점 만점에 평균 3.51점으로 비교적 낮은 수준이었는데, 이는 대상자가 임상적 우울군이 아니라 지역사회에 거주하는 기혼 여성이었기 때문으로 볼 수 있으며, 점수 분포가 낮아 변수 간 관련성의 크기가 다소 과소 추정되었을 가능성을 고려하여 해석해야 한다.

이를 바탕으로 폐경 여성의 우울을 예방하고 관리하기 위해서는 건강상태를 부정적으로 인식하는 여성을 조기에 확인하고, 폐경으로 인한 신체적 변화와 건강문제를 구체적으로 사정하여 개별적인 건강관리 요구에 맞는 중재를 제공할 필요가 있다. 또한 대상자의 건강상태와 생활환경을 고려한 실천 가능한 건강행위를 지속할 수 있도록 지원함으로써 건강관리 역량을 향상시키고 우울 예방 및 정신건강 증진에 기여할 수 있을 것이다.

부부동반활동의 경우 상관관계 분석에서는 우울과 미약한 음의 상관관계를 보였으나, 인구사회학적 특성과 주관적 건강상태를 통제한 최종 회귀모형에서는 독립적인 관련성이 확인되지 않았다. 이러한 결과는 부부가 함께하는 활동이 긍정적인 부부관계와 관련된다는 선행연구[14,47]와 비교할 때, 기혼 폐경 여성의 우울을 설명하는 데 부부동반활동의 단순 빈도만으로는 한계가 있음을 시사한다. 즉, 배우자와 함께 활동하는 빈도가 높더라도 그 활동이 정서적 교류, 관계 만족 및 상호 지지 경험으로 이어지지 않는다면 우울 감소와 직접적으로 연결되지 않을 수 있다[14,48]. 특히 부부동반활동과 주관적 건강상태 간에 유의한 정적 상관관계가 확인된 점을 고려할 때, 부부동반활동과 우울 간의 단순 상관관계에는 개인의 건강 인식이나 사회경제적 특성이 일부 관련되어 있을 가능성이 있다. 따라서 부부동반활동의 정신건강 효과를 평가할 때에는 활동 빈도와 함께 배우자와의 상호작용, 활동에 대한 만족도 및 활동 과정에서 경험하는 정서적 지지를 고려할 필요가 있다.

부부동반활동의 하위 영역별 추가 분석 결과, 영화·공연·스포츠 관람은 우울과 유의한 정적 관련성을 보였으나, 산책·조깅·등산·운동, 사회봉사·공동체 참여, 시댁 가족 만나기 및 친정 가족 만나기는 유의한 관련성이 없었다. 이러한 결과는 부부동반활동과 우울의 관계가 활동 유형에 따라 다르게 나타날 수 있음을 보여준다. 특히 영화·공연·스포츠 관람 빈도가 높을수록 우울 수준이 높게 나타났으나, 이를 해당 활동이 우울을 증가시키는 것으로 해석하는 데에는 주의가 필요하다. 본 연구는 횡단적 자료를 이용하였으므로 활동과 우울 간의 시간적 선후관계를 확인할 수 없으며, 우울 수준이 높은 여성이 신체적·심리적 부담이 비교적 적은 관람 중심의 여가활동에 더 자주 참여했을 가능성도 배제할 수 없다[19]. 또한 본 연구에서는 활동 과정에서의 배우자와의 상호작용이나 정서적 만족도를 측정하지 않았으므로, 관람활동의 빈도만으로 우울과의 관련성을 충분히 설명하기에는 한계가 있다.

산책·조깅·등산·운동이 우울에 유의한 관련성을 보이지 않은 결과는 신체활동이 스트레스 완화와 자기효능감 향상을 통해 정신건강 증진에 기여한다는 선행연구[44]와 차이가 있다. 다만 본 연구의 해당 문항은 개인의 신체활동이 아니라 배우자와 함께 수행한 신체활동의 빈도를 측정한 것이므로, 개인 수준의 신체활동과 우울의 관련성을 보고한 선행연구와 직접 비교하는 데에는 한계가 있다. 본 연구에서는 부부가 함께 수행한 신체활동의 빈도만을 측정하였으며 활동의 강도, 지속시간, 규칙성 및 만족도는 반영하지 못하였다. 또한 사회봉사·공동체 참여와 가족 만나기 역시 관계의 질이나 활동 과정에서 경험하는 정서적 지지와 부담에 따라 그 의미가 달라질 수 있다. 이러한 결과를 종합하면, 부부동반활동과 우울 간의 관련성을 보다 구체적으로 파악하기 위해서는 활동 참여 여부나 빈도뿐 아니라 활동의 질, 참여 경험 및 배우자와의 상호작용을 함께 고려해야 한다[48]. 간호학적 측면에서도 폐경 여성의 사회적·부부 활동을 사정할 때 단순한 활동 빈도보다는 활동 과정에서 경험하는 정서적 만족, 관계적 부담 및 지지 경험을 함께 확인하는 것이 필요하다.

이러한 맥락에서 부부동반활동은 배우자와 함께 활동하는 행위 자체보다 폐경기 변화에 함께 대처하고 정서적 지지를 주고받는 과정의 측면에서 고려해야 한다. 부부가 함께 정서적 지지를 주고받는 긍정적인 상호작용은 개인 및 관계적 차원의 이점과 관련되며[48], 부부를 대상으로 한 연구에서도 이러한 긍정적인 상호작용이 결혼만족도와 관련되는 것으로 나타났다[49]. 따라서 폐경 여성 대상 간호중재에서는 배우자를 건강관리의 지지 자원으로 포함하고, 부부가 폐경기 변화에 대한 정보를 공유하며 건강문제에 함께 대처할 수 있도록 지원해야 한다[50]. 또한 활동 횟수의 증가를 목표로 하기보다 부부의 의사소통과 정서적 지지를 촉진하고, 대상자의 신체 상태와 선호를 반영한 공동 활동 프로그램을 개발하여 실제 간호현장에 적용할 수 있는 방안을 마련할 필요가 있다.

본 연구에는 몇 가지 제한점이 있다. 첫째, 횡단적 연구 설계로 인해 주관적 건강상태, 부부동반활동 및 우울 간의 시간적 선후관계와 인과관계를 명확히 규명하기 어렵다. 둘째, 2차 자료를 활용하였기 때문에 부부동반활동 과정에서의 상호작용의 질, 활동에 대한 만족도, 참여의 자발성 및 활동 경험의 구체적 맥락을 충분히 반영하지 못하였다. 또한 본 연구에서는 여성가족패널조사의 부부동반활동 문항을 활용하여 배우자와 함께 수행하는 활동의 전반적인 수준을 반영하는 지표로 부부동반활동 총점을 산출하였다. 그러나 부부동반활동은 여가활동, 신체활동, 사회참여 및 가족교류 등 활동 유형별 특성이 서로 다르므로 총점을 각 활동 유형의 특성을 모두 반영하는 단일한 구성개념으로 해석하는 데에는 제한이 있다. 이에 본 연구에서는 총점 분석과 함께 활동 유형별 추가 분석을 실시하여 이러한 한계를 보완하고자 하였다. 따라서 부부동반활동 총점은 배우자와 함께 수행하는 활동의 전반적인 수준을 나타내는 지표로 해석할 필요가 있으며, 향후 연구에서는 활동 유형별 특성을 고려한 분석과 함께 부부동반활동 측정도구의 구성타당도를 검증하고, 활동 과정에서의 상호작용의 질, 활동에 대한 만족도 및 참여의 자발성을 함께 고려하여 부부동반활동과 우울 간의 관계를 보다 심층적으로 규명할 필요가 있다. 셋째, 본 연구는 배우자와 함께 거주 중인 기혼 폐경 여성만을 대상으로 하였으므로, 미혼, 동거, 별거, 이혼 등 다양한 혼인상태의 폐경 여성에게 연구 결과를 일반화하는 데 제한이 있다. 넷째, 본 연구에서는 단일 문항으로 측정된 주관적 건강상태를 연속형 변수로 처리하여 분석하였다. 이러한 방식은 선행연구에서도 활용되고 있으나, 리커트 척도를 연속형 변수로 간주하는 데에는 학문적 논의가 존재하므로 결과 해석에 주의가 필요하다. 마지막으로, 본 연구에서는 여성가족패널조사의 복합표본설계를 반영한 표본가중치와 층화 및 군집 정보를 분석에 적용하지 않았다. 따라서 조사설계에 따른 표준오차와 분산이 충분히 반영되지 않았을 가능성이 있으며, 연구 결과를 우리나라의 전체 기혼 폐경 여성에게 일반화하는 데에는 신중해야 한다. 향후 연구에서는 복합표본설계를 반영한 분석을 통해 본 연구 결과의 재현성과 일반화 가능성을 확인할 필요가 있다.

본 연구는 이러한 제한점에도 불구하고 기혼 폐경 여성의 우울과 관련하여 주관적 건강상태와 부부동반활동을 함께 고려하고, 각 요인의 상대적 관련성을 확인하였다는 점에서 의의가 있다. 특히 주관적 건강상태는 인구사회학적 특성을 통제한 이후에도 우울과 유의하게 관련된 주요 요인으로 확인되어, 폐경 여성의 정신건강 관리에서 자신의 건강상태에 대한 인식을 함께 평가할 필요성을 제시하였다. 또한 부부동반활동은 총점 수준에서는 우울과 독립적인 관련성이 확인되지 않았으며, 하위 영역 중 영화·공연·스포츠 관람만 우울과 유의한 정적 관련성을 보였다. 이는 부부동반활동의 빈도만으로 정신건강 효과를 설명하기 어렵고, 활동의 유형과 질, 참여 동기, 배우자와의 상호작용 및 활동 과정에서 경험하는 정서적 만족을 함께 고려할 필요가 있음을 보여준다. 따라서 향후 종단연구를 통해 주관적 건강상태, 부부동반활동 및 우울 간의 시간적·인과적 관계를 검증하고, 부부동반활동의 질적 측면을 반영한 측정과 분석이 이루어질 필요가 있다. 나아가 폐경 여성의 건강 인식을 개선하고 배우자의 정서적 지지와 공동 대처를 강화할 수 있는 간호중재 프로그램을 개발하여 그 효과를 검증해야 할 것이다. 이러한 연구의 축적은 기혼 폐경 여성의 정신건강 증진과 우울 예방을 위한 근거 기반 간호중재 전략 마련에 기여할 수 있을 것이다.

## Figures and Tables

**Figure 1.**
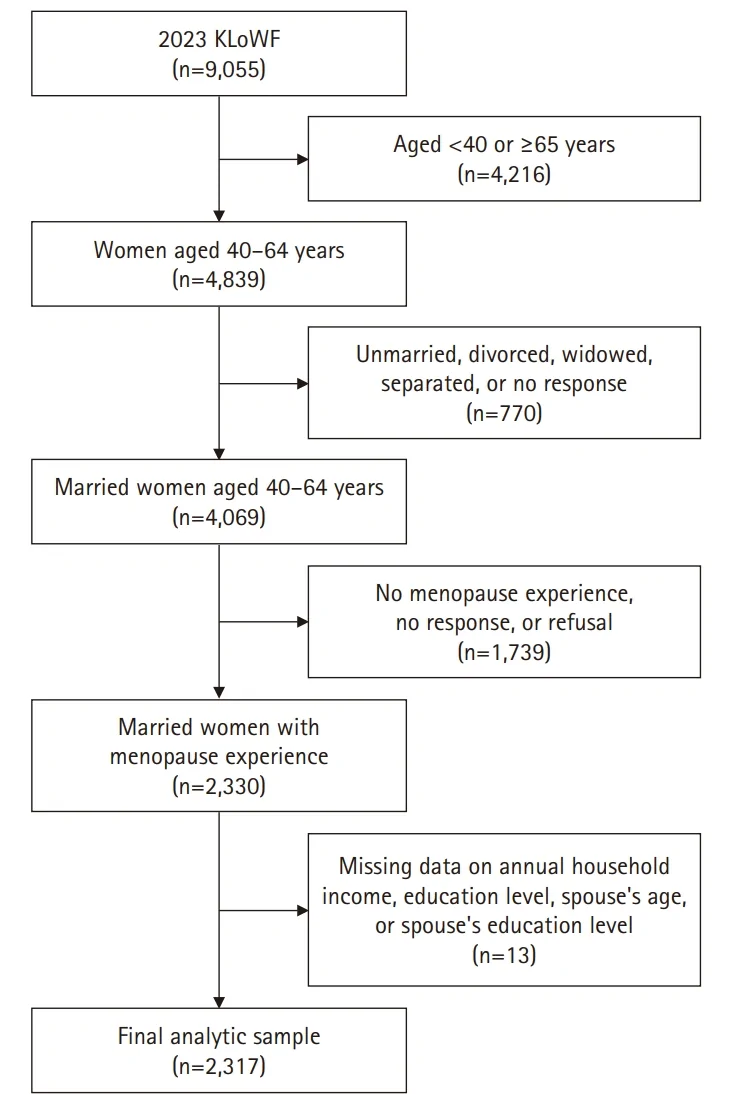
Flowchart of participant selection for the analytic sample. KLoWF: Korean Longitudinal Survey of Women and Families.

**Table 1.** Participant characteristics (N=2,317)

Variable	Category	n (%)	Mean±SD or median (IQR)
Age (year)	40–49	129 (5.6)	57.12±4.64
	50–59	1,367 (59.0)	
	60–64	821 (35.4)	
Education level	≤Middle school	449 (19.4)	
	High school	1,229 (53.0)	
	≥College	639 (27.6)	
BMI (kg/m^2^)	<18.5	40 (1.7)	23.30±2.70
	18.5–22.9	1,103 (47.6)	
	23.0–24.9	673 (29.1)	
	≥25.0	501 (21.6)	
Perceived body image	Thin	189 (8.2)	
	Normal	1,388 (59.9)	
	Obese	740 (31.9)	
Vigorous physical activity in the past week (day)	0	1,497 (64.6)	
	1–2	196 (8.5)	
	3–4	329 (14.2)	
	≥5	295 (12.7)	
Spouse’s age (year)	40–49	77 (3.3)	60.26±5.61
	50–59	923 (39.8)	
	60–69	1,222 (52.8)	
	≥70	95 (4.1)	
Spouse’s education level	≤Middle school	363 (15.7)	
	High school	1,063 (45.9)	
	≥College	891 (38.4)	
Annual household income^†^ (10,000 KRW)	Q1	577 (24.9)	6,000 (3,720–8,180)
	Q2	529 (22.8)	
	Q3	632 (27.3)	
	Q4	579 (25.0)	
Marital satisfaction	Dissatisfied	42 (1.8)	6.84±1.38
	Moderate	1,463 (63.1)	
	Satisfied	812 (35.1)	

BMI: body mass index; IQR: interquartile range; KRW: Korean won. At the 2023 annual average exchange rate of 10,000 KRW ≈ 7.7 US dollars.

† Q1–Q4 denote quartiles of annual household income, from the lowest (Q1) to the highest (Q4).

**Table 2.** Levels of subjective health status, spousal shared activities, and depression (N=2,317)

Variable	Mean±SD	Possible range	Actual range
Subjective health status	3.38±0.74	1–5	1–5
Spousal shared activities	6.88±2.50	5–25	5–25
Depression	3.51±4.40	0–30	0–30

**Table 3.** Depression by participant characteristics (N=2,317)

Variable	Category	Mean±SD	F or Welch’s F (*p*)	Post hoc comparison
Age (year)	40–49^a^	3.81±4.70	2.06 (.127)	
	50–59^b^	3.36±4.27		
	60–64^c^	3.72±4.56		
Education level	≤Middle school^a^	4.27±4.80	7.69 (<.001)	a>b,c^‡^
	High school^b^	3.39±4.31		
	≥College^c^	3.21±4.22		
BMI (kg/m^2^)	<18.5^a^	5.08±6.89	4.72 (.003)	d>b^‡^
	18.5–22.9^b^	3.18±4.10		
	23.0–24.9^c^	3.63±4.59		
	≥25.0^d^	3.95±4.48		
Perceived body image	Thin^a^	4.22±5.19	2.50 (.083)	
	Normal^b^	3.38±4.41		
	Obese^c^	3.58±4.16		
Vigorous physical activity in the past week (day)	0 days^a^	3.65±4.48	3.81 (.010)	a>c^‡^
	1–2^b^	3.18±3.98		
	3–4^c^	2.88±3.97		
	≥5^d^	3.72±4.69		
Annual household income^†^	Q1^a^	4.54±5.10	18.41 (<.001)	a>b,c,d; b,c>d^‡^
	Q2^b^	3.55±4.53		
	Q3^c^	3.32±4.19		
	Q4^d^	2.66±3.47		
Marital satisfaction	Dissatisfied^a^	8.02±6.39	44.05 (<.001)	a>b>c^‡^
	Moderate^b^	3.92±4.69		
	Satisfied^c^	2.55±3.37		

BMI: body mass index. Superscript letters (a, b, c, d) identify the groups compared in post hoc tests. Welch’s analysis of variance was used when the homogeneity-of-variance assumption was not met. Post hoc tests were conducted only when the between-group difference was significant.

† Q1–Q4 denote quartiles of annual household income, from the lowest (Q1) to the highest (Q4).

‡ Games-Howell test.

**Table 4.** Correlations among study variables (N=2,317)

Variable	r (*p*)		
	Subjective health status	Spousal shared activities	Depression
Subjective health status	1		
Spousal shared activities	.12 (<.001)	1	
Depression	–.35 (<.001)	–.05 (.017)	1

r denotes the Pearson correlation coefficient.

**Table 5.** Factors associated with depression among married postmenopausal women (N=2,317)

Variable	Category	Model 1				Model 2				Model 3			
		B	SE	β	t (*p*)	B	SE	β	t (*p*)	B	SE	β	t (*p*)
(Constant)		4.55	0.27		17.18 (<.001)	10.15	0.45		22.58 (<.001)	9.94	0.49		20.43 (<.001)
Age (reference, 60–64 years)	40–49	0.92	0.42	.05	2.18 (.029)	1.02	0.40	.05	2.51 (.012)	0.99	0.41	.05	2.45 (.014)
	50–59	0.31	0.21	.03	1.45 (.148)	0.39	0.20	.04	1.93 (.053)	0.39	0.20	.04	1.94 (.052)
Educational level (reference, ≤Middle school)	High school	–0.40	0.26	–.05	–1.56 (.118)	–0.17	0.25	–.02	–0.68 (.495)	–0.20	0.25	–.02	–0.79 (.428)
	≥College	–0.33	0.30	–.03	–1.09 (.276)	–0.07	0.29	–.01	–0.22 (.823)	–0.11	0.29	–.01	–0.38 (.701)
Annual household income^†^ (reference, Q1)	Q2	–0.93	0.27	–.09	–3.49 (<.001)	–0.67	0.25	–.06	–2.63 (.009)	–0.68	0.25	–.07	–2.67 (.008)
	Q3	–1.06	0.26	–.11	–4.03 (<.001)	–0.75	0.25	–.08	–3.01 (.003)	–0.77	0.25	–.08	–3.06 (.002)
	Q4	–1.56	0.28	–.15	–5.59 (<.001)	–1.18	0.27	–.12	–4.42 (<.001)	–1.21	0.27	–.12	–4.50 (<.001)
BMI (reference, 18.5–22.9)	<18.5	1.80	0.69	.05	2.62 (.009)	1.08	0.66	.03	1.63 (.102)	1.08	0.66	.03	1.64 (.100)
	23.0–24.9	0.38	0.21	.04	1.79 (.074)	0.30	0.20	.03	1.48 (.140)	0.30	0.20	.03	1.49 (.137)
	≥25	0.57	0.24	.05	2.42 (.015)	0.16	0.23	.02	0.69 (.488)	0.16	0.23	.02	0.72 (.470)
Marital satisfaction (reference, Moderate)	Dissatisfied	4.02	0.67	.12	6.01 (<.001)	3.07	0.64	.09	4.78 (<.001)	3.11	0.64	.09	4.85 (<.001)
	Satisfied	–1.18	0.19	–.13	–6.19 (<.001)	–0.71	0.18	–.08	–3.84 (<.001)	–0.73	0.19	–.08	–3.94 (<.001)
Subjective health status		–				–1.81	0.12	–.31	–15.06 (<.001)	–1.82	0.12	–.31	–15.10 (<.001)
Spousal shared activities		–				–				0.04	0.04	.02	1.13 (.257)
Sensitivity analyses^‡^													
Additional adjustment for vigorous physical activity										0.04	0.04	.03	1.20 (.230)
Marital satisfaction excluded										0.01	0.04	.01	0.32 (.748)
F (*p*)		13.40 (<.001)				31.03 (<.001)				28.91 (<.001)			
R^2^		.07				.15				.15			
Adjusted R^2^		.06				.14				.14			
Change in R^2^ (*p*)		.07 (<.001)				.08 (<.001)				.00 (.257)			

Model 1 included control variables only (age, education level, annual household income, BMI, and marital satisfaction); Model 2 additionally included subjective health status; Model 3 additionally included spousal shared activities. BMI: body mass index.

† Q1–Q4 denote annual household income quartiles, from the lowest (Q1) to the highest (Q4). B and β denote unstandardized and standardized regression coefficients, respectively.

‡ Sensitivity analyses used Model 3 as the base model; the coefficients shown are for spousal shared activities. The first sensitivity model additionally adjusted for vigorous physical activity using three dummy variables (1–2 days, 3–4 days, and ≥5 days), with 0 days as the reference category. The second sensitivity model excluded marital satisfaction.

## Supplementary materials

Further details on supplementary materials are presented online (available at https://doi.org/10.4069/whn.2026.09.15).

Supplementary Table 1. Associations between spousal shared activity subdomains and depression (N=2,317)
