# Supplementary material for: Associations of subjective health status and spousal shared activities with depression among married postmenopausal women: a correlational study using data from the ninth wave of the Korean Longitudinal Survey of Women and Families
Source: Womens Health Nurs. 2026 Sep 29;32(3):269–82. [Article in Korean] doi: 10.4069/whn.2026.09.15 (PMC13654673; doi:10.4069/whn.2026.09.15)
Supplement: Supplementary file 1 [file whn-2026-09-15-Supplementary-Table-1.pdf]

Supplementary Table 1. Associations between spousal shared activity subdomains and depression (N=2,317)

Subdomains of spousal shared activities	B	SE	β	t (*p*)
Watching movies, performances, and sports events	0.65	0.18	.08	3.66 (<.001)
Walking, jogging, hiking, and exercising	–0.17	0.09	–.04	–1.93 (.054)
Volunteering and community participation	0.13	0.15	.02	0.86 (.391)
Meeting the husband's family	0.18	0.16	.03	1.12 (.265)
Meeting the wife's family	–0.27	0.18	–.04	–1.56 (.119)

B and β denote unstandardized and standardized regression coefficients, respectively. The analysis was adjusted for age, educational level, annual household income, body mass index, marital satisfaction, and subjective health status.
